# Novel Virus Influenza A (H1N1sw) in South-Eastern France, April-August 2009

**DOI:** 10.1371/journal.pone.0009214

**Published:** 2010-02-17

**Authors:** Antoine Nougairède, Laetitia Ninove, Christine Zandotti, Nicolas Salez, Karine Mantey, Noémie Resseguier, Céline Gazin, Didier Raoult, Rémi N. Charrel, Xavier de Lamballerie

**Affiliations:** 1 Unité Mixte de Recherche 190: Unité des Virus Emergents, Université de la Méditerranée et Institut de Recherche pour le Développement, Marseille, France; 2 Laboratoire de Virologie, Pôle Hospitalier de Microbiologie et Maladies Infectieuses (Assistance Publique – Hôpitaux de Marseille), Marseille, France; 3 South Interregional Epidemiology Unit, French Institute for Public Health Surveillance, Marseille, France; Institute of Molecular and Cell Biology, Singapore

## Abstract

**Background:**

In April 2009, the first cases of pandemic (H1N1)-2009 influenza [H1N1sw] virus were detected in France. Virological surveillance was undertaken in reference laboratories of the seven French Defence Zones.

**Methodology/Principal Findings:**

We report results of virological analyses performed in the Public Hospitals of Marseille during the first months of the outbreak. (i) Nasal swabs were tested using rapid influenza diagnostic test (RIDT) and two RT-PCR assays. Epidemiological characteristics of the 99 first suspected cases were analyzed, including detection of influenza virus and 18 other respiratory viruses. During three months, a total of 1,815 patients were tested (including 236 patients infected H1N1sw virus) and distribution in age groups and results of RIDT were analyzed. (ii) 600 sera received before April 2009 and randomly selected from in-patients were tested by a standard hemagglutination inhibition assay for antibody to the novel H1N1sw virus. (iii) One early (May 2009) and one late (July 2009) viral isolates were characterized by sequencing the complete hemagglutinine and neuraminidase genes. (iiii) Epidemiological characteristics of a cluster of cases that occurred in July 2009 in a summer camp were analyzed.

**Conclusions/Significance:**

This study presents new virological and epidemiological data regarding infection by the pandemic A/H1N1 virus in Europe. Distribution in age groups was found to be similar to that previously reported for seasonal H1N1. The first seroprevalence data made available for a European population suggest a previous exposure of individuals over 40 years old to influenza viruses antigenically related to the pandemic (H1N1)-2009 virus. Genomic analysis indicates that strains harbouring a new amino-acid pattern in the neuraminidase gene appeared secondarily and tended to supplant the first strains. Finally, in contrast with previous reports, our data support the use of RIDT for the detection of infection in children, especially in the context of the investigation of grouped cases.

## Introduction

The first cases of the new H1N1 pandemic influenza virus (H1N1sw), in metropolitan France, were detected in April 2009 in patients returning from Mexico. Systematic analysis of suspected cases [1] was undertaken and the virus was identified, using molecular methods, in the Public Hospital virology “Level A” laboratories of the seven French Defence Zones. Accordingly, samples from the Southern Defence Zone (a large geographical region encompassing Corsica and the Mediterranean costal zone from the Spanish border to the Italian border with approximately 8 million inhabitants), were received and analysed in our department, at the Virology Level A laboratory of the Public Hospitals of Marseille.

The current study refers to samples received between the end of April and the end of August 2009. During the first period (until mid-July), samples were systematically collected using strict and identical criteria, mainly based either on the presence of an acute respiratory illness and recent travel history in an affected area, or on contact with a confirmed or suspected case. During the second period, biological confirmation of suspected cases was no longer required and criteria used for requesting biological diagnosis (grouped cases, severe or atypical presentations, pre-existing condition etc.) were more heterogeneous.

Here, we present the results of virological analyses performed during the first three months that followed the introduction of the novel H1N1sw pandemic influenza variant in metropolitan France. This included the detection and characterization of influenza viruses, the evaluation of rapid Influenza detection tests (RIDTs) detection of the H1N1sw pandemic variant, the detection of other respiratory viruses and the investigation of grouped cases. In addition, the distribution of specific antibody to the new virus was investigated according to age groups in a sample of 600 individuals. Altogether, these data shed new light on the determinants of the epidemiological distribution of viral infection in the French population.

## Methods

### Samples Collected between April 25^th^, 2009 and August 31^st^, 2009

The biological material studied here was used only for standard diagnostic procedures following physicians' prescriptions (no specific sampling, no modification of the sampling protocol). Analysis of data was performed using an anonymized database. Following local regulations, this procedure did not require a specific consent from patients.

**Figure 1:**
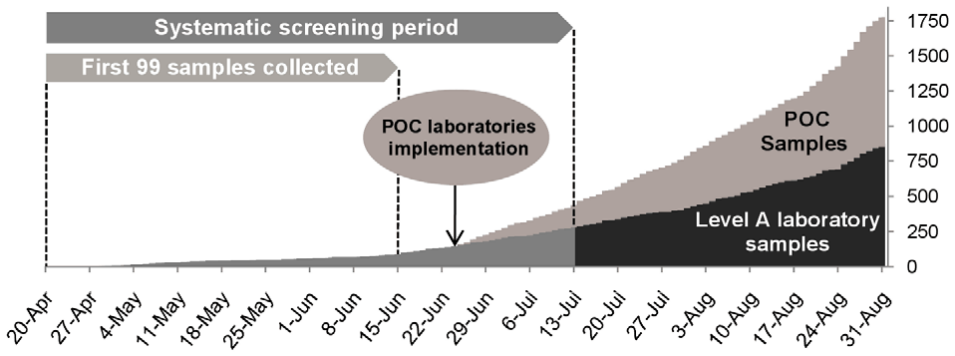
Samples tested from April to August 2009. Panel A corresponds to Level A laboratory samples (N = 99) tested between April 25^th^ 2009 and June 15^th^ 2009. Panel B corresponds to Level A laboratory samples (N = 280) tested between April 25^th^ 2009 and July 13^th^ 2009. Panel C corresponds to all samples (N = 1,815) tested between April 25^th^ 2009 and August 31^st^ 2009, including Point of Care (POC) samples.

Nasal swabs received between April 25^th^, 2009 and August 31^st^, 2009 were included in the study (see figure 1). Until mid-July 2009, criteria used for sample collection were strict and identical for all patients: a possible case was defined as a person with acute respiratory illness (defined as the occurrence of fever (>38°C) or myalgia or asthenia and at least one respiratory symptom (cough or dyspnoea) and a history of travel in an affected area or a history of close contact with a confirmed or possible case one day to seven days before the onset of symptoms. In order to capture cases from previously undetected chains of transmission, clusters of acute respiratory illness defined as at least three cases in a week in closed communities were also to be notified [1]. During the subsequent period, criteria used for requesting biological diagnosis were modified. The biological confirmation of suspected cases was no longer systematic, an increasing number of patients with influenza-like presentation and no history of travel abroad or contact with documented cases was tested, including grouped cases, severe or atypical presentations, patients with pre-existing condition etc. In addition, a Point Of Care (POC) strategy was applied from June 23^rd^ for the Public Hospitals of Marseilles [2] (see figure 1).

Accordingly, three different panels were studied: (i) The first 99 samples collected until June 15^th^, 2009 using the systematic criteria for suspected cases reported above in the Southern Defence Zone (Panel A).(ii) All 280 samples collected from April 25^th^, 2009 to July 13^th^, 2009 using the systematic criteria for suspected cases reported above in the Southern Defence Zone (Panel B). (iii) The total of all 1,815 samples collected by our group during the study period (Panel C, see figure 1).

All samples were tested for the presence of Influenza A virus using a pan-influenza A real time PCR technique as described elsewhere [3] and a second real time PCR specific for the new H1N1 variant [1]. In addition, the first 99 samples collected (Panel A) were also tested by real time PCR techniques for the presence of a panel of 18 different respiratory viral pathogens [3], [4], [5], [6], [7], [8], [9], [10], [11], [12], [13], [14], [15], [16] (see table 1).

**Table 1:** Etiology of viral respiratory infections in Panel A.

Viral etiology	Number	Country	Detection protocol
Influenza virus A virus H1N1sw 2009	15	UK (1), USA (4), Spain (1), Canada (3), Mexico (2), France (4)	*Ninove L, Vector Borne Zoonotic Dis. 2009*
Influenza virus A virus H3N2	2	UK (1), USA (1)	*Van Elden L.J.R, J Clin Microbiol. 2001*
Influenza virus B virus	0	/	*Van Elden L.J.R, J Clin Microbiol. 2001*
Influenza virus C virus	0	/	*Gouarin S, J Med Virol. 2008*
Rhinovirus	5*	USA (1), France (3), Japan (1)	*Garbino J, Am J Respir Crit Care Med. 2004*
Metapneumovirus	2*	UK (1), France (1)	*Mackay IM, J Clin Microbiol. 2003*
Respiratory Syncytial Virus A/B	0	/	*Van Elden L.J.R, J Clin Microbiol. 2003*
Human Coronavirus 229E	1*	Canada (1)	*Van Elden L.J.R, J Infect Dis 2004*
Human Coronavirus OC43	2	USA (2)	*Van Elden L.J.R, J Infect Dis 2004*
Human Coronavirus NL63	0*	/	*Tiveljung-Lindell A, J Med Virol. 2009*
Human Coronavirus KU1	0	/	*Tiveljung-Lindell A, J Med Virol. 2009*
Enterovirus	1	France (1)	*Dierssen U, J Clin Virol. 2008*
Parechovirus	0	/	*Benschop K, J Clin Virol 2008*
Polyomavirus KI	0	/	*Lindau C, J Clin Virol. 2009*
Polyomavirus WU	1*	USA (1)	*Lindau C, J Clin Virol. 2009*
Parainfluenza virus 1/2/3/4	5	USA (2), Mexico (2), Unknow (1)	*Tong S, J Clin Microbiol. 2008*
Bocavirus	4	France (2), Australia (1), Unknow (1)	*Allander T, Clin Infect Dis. 2007*
Cytomegalovirus	0	/	*Griscelli F, J Clin Microbiol. 2001*
Human Coronavirus 229E + Polyomavirus WU	1	USA (1)	*/*
Human Coronavirus NL63 + Rhinovirus	1	Mexico (1)	/
Metapneumovirus + Polyomavirus WU	1	Mexico (1)	/
Negative samples	58	UK (2), USA (15), Spain (3), Canada (4), Mexico (14), France (15), Unknown (5)	/
**Total number**	99	/	/

The etiological agent, the number of cases, the geographical origin of patients returning from abroad and the references for the methods used for molecular diagnosis are indicated.

*: see also multiple infections.

### Viral Loads, RIDTs

During the period of study, 1808 samples received in our department were tested using the Directigen “BD EZ A+B” (Becton Dickinson & company) RIDT for the detection of influenza A and B antigens.

The H1N1sw viral load was investigated in 41 positive samples by re-extracting samples and amplifying them simultaneously using a probe-based real time RT-PCR technique [1], and quantified by serial dilutions of a positive control based on synthetic RNA. The relationship between viral load and RIDT result was then analysed.

### Investigation of Virus Infection in a Summer Camp

The investigation protocol presented here was elaborated by the French “Institut National de Veille Sanitaire” and validated by the Ethic Committee “ CPP Ile-de-France IX”.

In July 2009, a cluster of cases in a summer camp in Barcelonnette (Alpes-de-Haute-Provence, France) was investigated. Case definition for analysis was as follows: possible cases were individuals with acute respiratory syndrome (coughing or dyspnoea) + a general presentation of viral infection (fever >38°C, or asthenia or myalgia); probable cases were possible cases who had a close contact with a confirmed case (in the period encompassing 2 days before and 7 days after the onset of the first symptom of this confirmed case; confirmed cases were possible or probable cases with microbiological confirmation (positive RT-PCR from nasal swab).

All 94 children (6 to 14 years old, median: 10) had arrived in the summer camp on July 20^th^. They were supervised by 28 adults (16 counsellors or members of the management team and 12 technical agents, *i.e.* kitchen and cleaning staff).

During the investigation process, one nasal swab could be sampled from 95% of probable cases and 85% of “non-cases”. Samples were submitted to H1N1sw detection using the same RT-PCR methods as reported above.

### Prevalence of Antibodies to the New H1N1 Variant

This research protocol was approved by the Departmental (IFR48) Ethic Committee and did not require patient consent. Only biological archival material was used (no specific sampling, no modification of sampling procedures). All information contained in databases was de-identified.

A collection of 3,000 sera received between January 2009 and March 2009 (before the detection of the first cases of H1N1sw infections on the French territory) in our laboratory for performing a variety of serological investigations was established. Six hundred sera distributed in 4 age groups (0–19, 20–39, 40–59 and >60) were randomly selected in this collection, until an equal number of 150 sera in each age group was reached.

Antibodies to the new H1N1sw virus were detected and quantified by the standard hemagglutination inhibition (HI) technique. The antigen was prepared from cell culture supernatant medium obtained following a seven-day propagation of strain OPYFLU-1 at high m.o.i. onto MDCK cells. Serial dilutions of heat-decomplemented serum (1/20–1/5,120), four viral hemagglutinating units and a suspension of human erythrocytes (group O, final concentration: 0.5%) were used in a final volume of 50 µL. In addition, a subgroup of 300 randomly selected human serum samples was tested using antigens from seasonal influenza viruses, *i.e.* one strain of seasonal H1N1 (Marseille-2007), and one strain of seasonal H3N2 (Marseille-2008).

### Sequence Analysis

The complete sequence of the hemagglutinin (HA) and neuraminidase (NA) genes of two different H1N1sw strains were analysed. Strain OPYFLU-1 was isolated from nasal swabs sampled from a young adult male patient returning from Mexico in early May 2009, following inoculation onto MDCK cells. Strain OPYFLU-58 was isolated from a case of autochthonous viral transmission (teenager, male) in late July. After RNA extraction from infected cell culture supernatant medium using the EZ1 Biorobot and the virus mini kit (both from Qiagen), one-step RT-PCR reactions were performed using the Access RT-PCR Core Reagents Kit (Promega Corporation, Madison, WI, USA) on TProfessional Standard Thermocycler (Biometra biomedizinische Analytik GmbH Goettingen Germany) and H1N1sw specific primers available upon request to the corresponding author [17] (WHO Genome Primers). PCR-fragments of 1,809 (HA gene) and 1,362 nucleotides (NA gene) were obtained and sequenced (Big Dye Terminator v1.1 Cycle Sequencing kit, Applied Biosystems, Foster City, CA,USA). Data from sequencing reactions were combined for analysis and edited using the Sequencher 4.7 software (Gene Codes Corporation).

Sequences were analysed and compared with H1N1sw 2009 HA and NA sequences available in databases at mid-October 2009. Complete HA and NA amino-acid sequences were aligned with ClustalX [18] and phylogenetic trees were built using nucleotide or amino-acids alignments with MEGA version 4.1 [19] using various methods (Neighbor-Joining, Maximum Parsimony and UPGMA) with 1,000 bootstrap replicates.

## Results

### First 99 Samples Collected from Suspected Cases in the Southern Defence Zone (Panel A)

**Figure 2:**
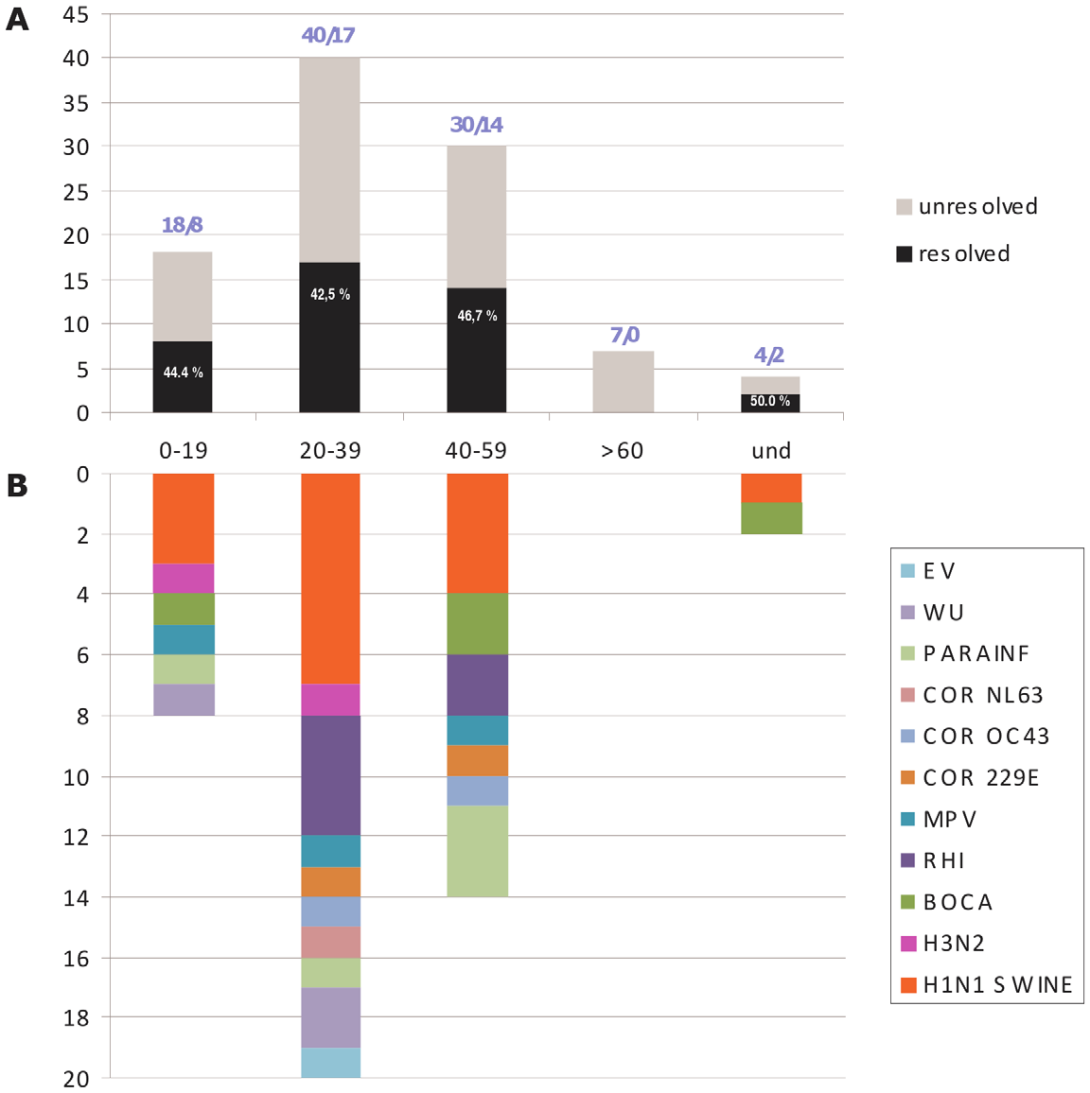
Etiology of viral respiratory infections in Panel A by age groups. Figure 2A shows the distribution in age groups of suspected cases tested/positive for H1N1sw. Figure 2B details the different etiologies in each age group. *: includes 3 co-infections. EV: enteroviruses; WU: WU polyomavirus; PARAINF: parainfluenza viruses 1/2/3/4; COR NL63: human coronavirus NL63; COR OC43: human coronavirus OC43; COR 229E: human coronavirus 229E; MPV: human metapneumovirus; RH: rhinoviruses; BOCA: bocaviruses; H3N2: influenza A virus H3N2; H1H1: influenza A virus H1N1sw. Und: undetermined.

All 99 samples were collected from symptomatic patients returning from abroad (Mexico (n = 20), USA (n = 27), Canada (n = 8), Japan (n = 1), Australia (n = 1), UK (n = 5), Spain (n = 4), unknown (n = 7), see table 1) or who had close contact with a patient returning from abroad (26 patients). The median age was 33 (range: 0–76 years) and the m/f sex ratio was 0.94. The distribution in age groups (see figure 2A) demonstrates that the majority of travellers were in the 20–60 years old age group with a limited number of patients under 20 and above 60. Most of the children tested were less than 5 years old.

Results are detailed in table 1 and show that in 41% of cases, one or several possible viral etiologic agents were identified. The pandemic influenza virus was found in 15% of cases but rhinoviruses, pneumoviruses, coronaviruses, enteroviruses, polyomaviruses, and parainfluenza viruses could also be identified. No case of multiple infection implicating influenza and another agent was detected. Among H1N1sw positive patients, the median age was 32 and the sex ratio was 0.75.

The distribution of respiratory viral pathogens detected in age groups is detailed in figure 2B. The percentage of etiological identification (including the percentage of H1N1sw detection) was similar in all age groups. Most of the cases of H1N1sw infection (11 out of 15, *i.e.* 73%) were found in the 20–60 age group (which included 70% of the samples studied), while 20% of cases were identified in the group of patients less than 20 years old (which included 18% of the samples studied).

### Samples Collected for the Documentation of Suspected Cases (Panels B and C)

**Figure 3:**
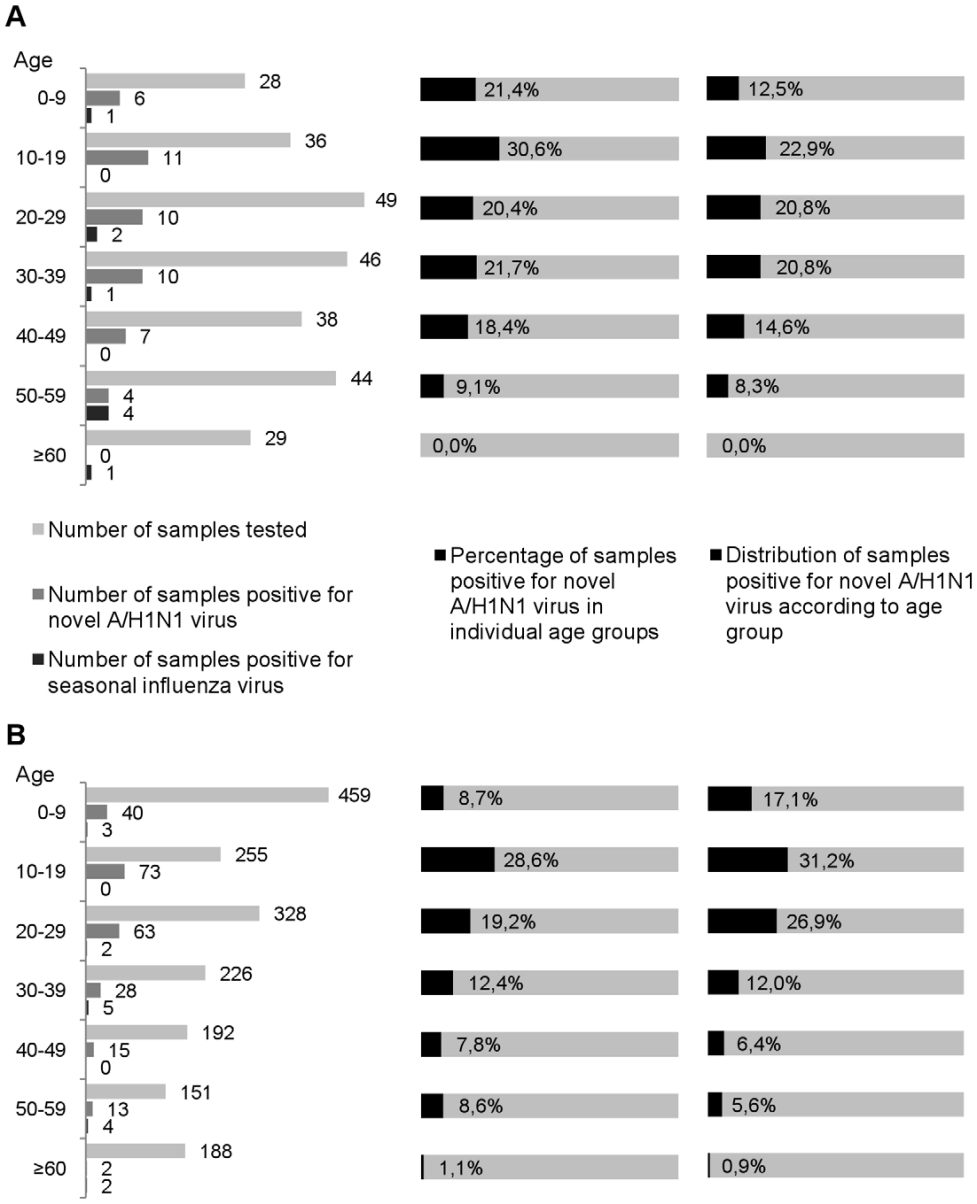
Distribution of cases in age groups for Panel B and C. Figure 3A and 3B show the distribution of cases in age groups for the Panel B and C respectively. The figure includes only patients whose age was known (270 patients from Panel B and 1799 in Panel C). The column on the left shows the number of samples tested, positive for H1N1sw or positive for seasonal H3N2 virus in each age group. The column in the middle shows the percentage of samples testing positive for H1N1sw in each age group. The column on the right shows the distribution of positives in the different age groups.

Panel B included 280 samples collected between April 25^th^, 2009 and July 13^th^, 2009 using the systematic criteria for suspected cases reported above. The sex ratio was 1.06 and the median age was 33 (range: 0–90 years), *i.e.* similar to that of panel A. The distribution in age groups is reported in figure 3A for 270 patients of known age and shows the lowest numbers under 10 years of age (10%) and over 60 (10%), and also a first readjustment compared with panel A: the number of samples tested in the 10–19 age group increased (13%) and the highest rate of positive H1N1sw diagnosis (above 30%) was observed in this group. Overall, 65% of H1N1sw cases were identified in patients 10–39 years old, explaining the decreased observed median age (25.5) amongst H1N1sw positive patients. The sex ratio in H1N1sw patients was 0.96.

**Figure 4:**
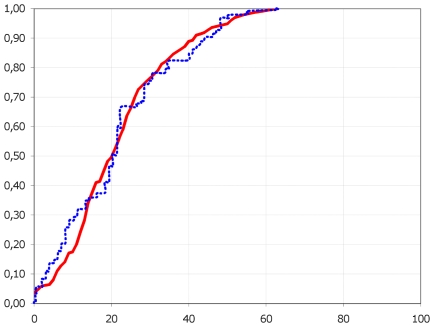
Empirical cumulative distribution of ages for patients with seasonal H1N1 or novel H1N1sw virus. We show the empirical cumulative distribution of ages for patients with seasonal H1N1 (blue) in New York State during the 2006–2007 and 2007–2008 influenza seasons and for H1N1sw (red) in Panel C.

Panel C included all 1,815 nasal swabs tested between April 25^th^ 2009 and August 31^st^ 2009 in our laboratory, received either from the general survey of the population within the Southern Defence Zone or from patients hospitalised in the Public Hospitals of Marseilles. The sex ratio was 1.03 and the median age was 24 (range: 0–98 years), *i.e.* lower than in panels A and B. The distribution in age groups (see figure 3B which includes 1,779 patients of known age) reveals that the number of tests performed for children under the age of 10 increased sharply (25%), and remained limited for patients over 60 years old (10%). Again, the highest rate of positive H1N1sw diagnosis was observed in the age group 10–19 years old. Approximately 50% of all infections were found in patients under the age of 20 (median age of H1N1sw positive patients: 21; sex ratio: 1.07) and very few cases (less than 1%) were identified in patients over 60 years old. This distribution is strikingly similar (see figure 4) to the picture of the distribution of H1N1 seasonal influenza reported by [20] in various geographical locations and periods of time, but also very different from the distribution reported by the same authors for H3N2 viruses (which included a significantly higher number of cases in the elderly).

Statistical analysis showed a different distribution of positives in different age groups: the number of cases was significantly lower in patients over 40 years old (Panel B, p = 0.005; Panel C, p<0.0001) compared with younger patients.

**Figure 5:**
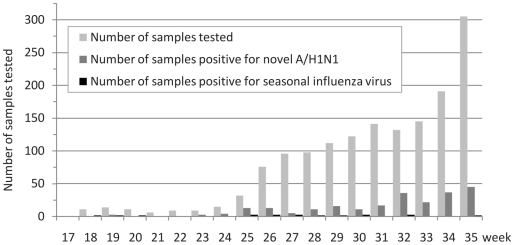
Weekly distribution of samples tested and samples positive for H1N1sw or seasonal H3N2 virus.

Figure 5 shows the evolution of the situation over time in Panel C. Interestingly, the proportion of influenza infections caused by seasonal H3N2 constantly decreased during the period of the study (final value <5%), but the absolute number of cases observed weekly remained roughly constant, suggesting that seasonal influenza circulated at low rate during this summer period (a phenomenon never observed previously and which may reflect the previous poor performance of the routine surveillance systems implemented for respiratory infections) and that this circulation was not markedly modified by the increasing number of cases of H1N1sw infections.

### Viral Loads, Results of RIDTs

**Figure 6:**
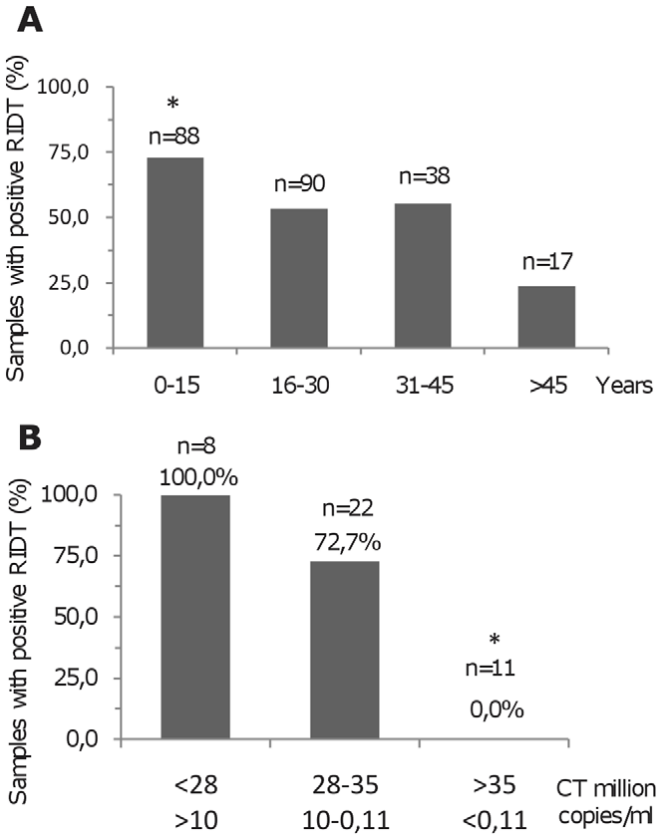
Results of RIDTs according to age groups and viral load. Figure 6A shows the distribution of positive RIDTs in age groups amongst 233 samples positive for H1N1sw based on RT-PCR techniques. Figure 6B shows the distribution of positive RIDTs according to viral load amongst 41 samples. *: p<0.001 (chi-square test); compared with all other samples.

Analysis of the results of RIDT for the detection of influenza A and B antigens showed that no false positive was identified (Specificity and Positive Predictive Value  = 100%), but false negative results were encountered. Accordingly, the relationship between age, viral load and result of RIDTs was investigated. First, amongst 233 samples positive for H1N1sw based on RT-PCR techniques, the distribution of positive RIDTs in age groups was examined (see figure 6A). This revealed an optimal sensitivity (∼75%) in patients younger than 15 (p<0.001, compared with other age groups) and a poor sensitivity in patients over 45 (<25%).

The relationship between viral load and RIDT result was then analysed (see figure 6B). This revealed that samples with high viral loads (>10 million copies/mL) could be constantly detected by the BD RIDT. The sensitivity of the RIDT test decreased with viral load and no positive result was obtained for samples with viral loads <0.11 million copies/mL. The relationship between viral load and results of RIDT was supported by statistical tests.

Overall the strong relationship between positive RIDTs and high viral loads on the one hand, and the group of patients in the age group 0–15 on the other hand, strongly suggests that viral excretion is more pronounced in children, in accordance with previous results obtained for seasonal influenza [21], [22].

### Investigation of Virus Infection in a Summer Camp

**Figure 7:**
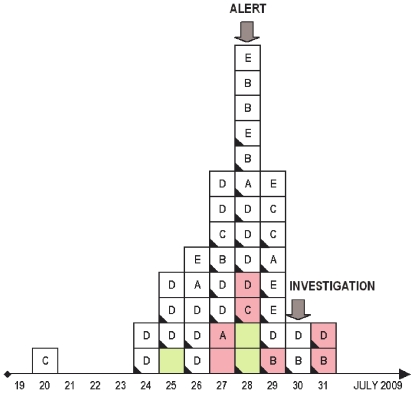
Probable and confirmed cases in a summer camp, July 2009. White cubes figure children; pink cubes figure counsellors/management team; yellow cubes figure technical staff. Letters indicate the group (see main text) and the cubes with a black corner indicate microbiological confirmation.

45 persons met the definition of probable or confirmed cases. They all reported coughing and 82% reported fever >38°C (see table 2). Thirty six cases were children (median age: 11; extremes: 8–13) and 9 were adults (median age: 22; extremes: 19–50). There was no significant difference in terms of age and sex between ill and non-ill children. The chronological onset of cases is represented in the epidemic curve (see figure 7). Children and counsellors had been distributed in different groups (A, B, C, D, E). All individuals in a given group were sharing daytime activities and were sleeping at night on the same floor in the main building of the camp, with the exception of few children from group C, including the index case, who shared the floor of teenagers of group D. The index case was retrospectively identified and occurred on the day of arrival of the children in the camp. No history of travel or previous contact with a suspected case could be identified for this child. The outbreak peak was reached eight days after the onset of the index case. An alert was then issued, and a case-management procedure was implemented, with physical separation between symptomatic and asymptomatic children (and adults). The investigation was performed 2 days after the alert in a period characterized by the rapid decline of the outbreak.

**Table 2:** Clinical symptoms observed in 45 probable and confirmed cases in a summer camp, July 2009.

Symptoms	N	%
Coughing	45	100
Fever	37	82
Asthenia	34	76
Headache	27	60
Myalgia	27	60
Sore throat	27	60
Shivering	17	38
Rhinitis	15	33
Nausea	6	13
Dyspnoea	5	11

The global attack rate was 38% in children, 37.5% in adults managing children, and 25% in technical agents. It was therefore similar in children and adults in close contact with them. However, this attack rate was different in the different groups varying from 19% in group A to 57.5% in group D. The latter group was constituted by teenagers (10–14 years old) which represented the majority of the secondary cases observed during the first days of the outbreak (see figure 7).

During the investigation process, one nasal swab could be sampled from 95% of probable cases and 85% of “non-cases”. Samples were submitted to H1N1sw detection using the same RT-PCR methods as reported above. Interestingly, the virus was detected in 7 of the 67 “non-cases” tested (10,4%). One was a child with fever and asthenia but without any respiratory symptoms. A telephone follow-up of the 6 remaining asymptomatic persons was organised. One child and one counsellor experienced coughing and fever by July 31^st^ and were included in data analysis (see figure 7). One week after sampling, two children had experienced isolated rhinitis, but two others remained totally asymptomatic. Finally, amongst the 7 “non-cases” tested, 2 became typical influenza cases, 3 had atypical presentations, and 2 remained completely asymptomatic.

### Prevalence of Antibodies to the New H1N1 Variant

**Figure 8:**
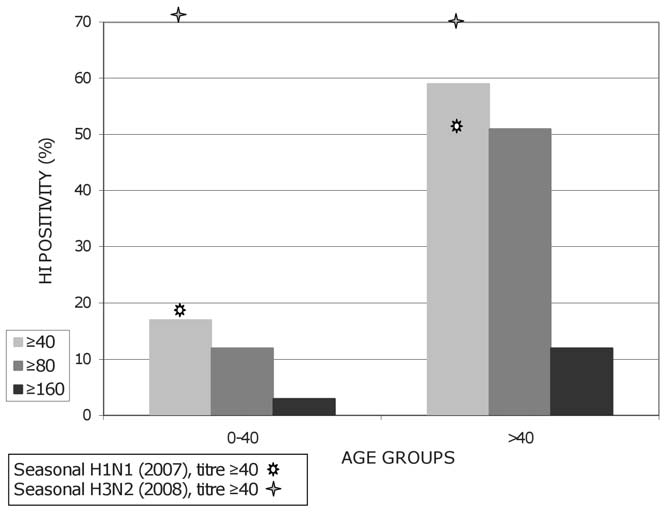
Prevalence of antibodies to H1N1sw and seasonal influenza viruses according to age. The prevalence of antibodies to H1N1sw is given for HI titres ≥1/40, ≥1/80 and ≥1/160. The prevalence of antibody to seasonal H1N1 (using a strain isolated in Marseille in 2007), and to seasonal H3N2 (using a strain isolated in Marseille in 2008) is given for HI titres ≥1/40.

The prevalence of antibodies to the new H1N1sw variant in patients under and above the age of 40 is shown in figure 8. The prevalence at different titres (≥1/40, ≥1/80, ≥1/160) is significantly lower in patients under the age of 40 (p<0. 0001). This distribution is different from that observed for antibodies to H1N1 and H3N2 seasonal viruses. In the case of seasonal H1N1, the prevalence of HI titre ≥1/40 is similar to that observed for H1N1sw amongst patients under the age of 40, but slightly lower in patients over 40. However, no statistical relationship could be identified between individual titres of antibodies to H1N1sw and seasonal H1N1. For seasonal H3N2, the prevalence of HI titre ≥40 is higher in both groups (with an important difference for patients under the age of 40). Again, no statistical relationship could be identified between individual titres of antibodies to H1N1sw and seasonal H3N2.

**Figure 9:**
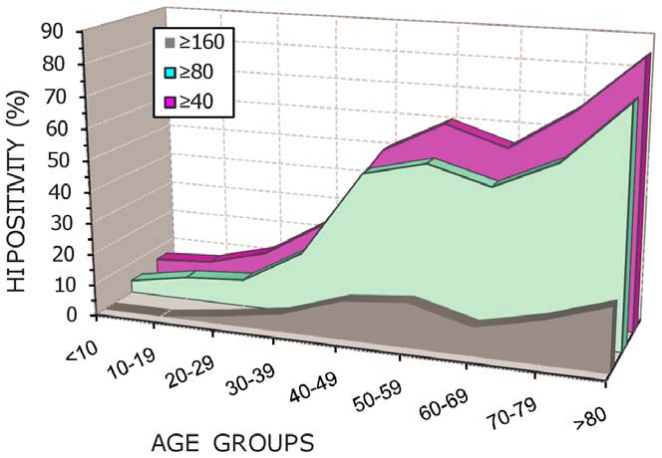
Prevalence of antibody to H1N1sw according to age groups. The prevalence is given for HI titres ≥1/40, ≥1/80 and ≥1/160.

Figure 9 shows a more detailed distribution of antibodies to H1N1sw in age groups. A similar age-dependent fluctuation of prevalence was observed for all HI titres, but it should be noted that the prevalence of titres ≥1/160 remains globally modest at all ages.

### Sequence Analysis

**Figure 10:**
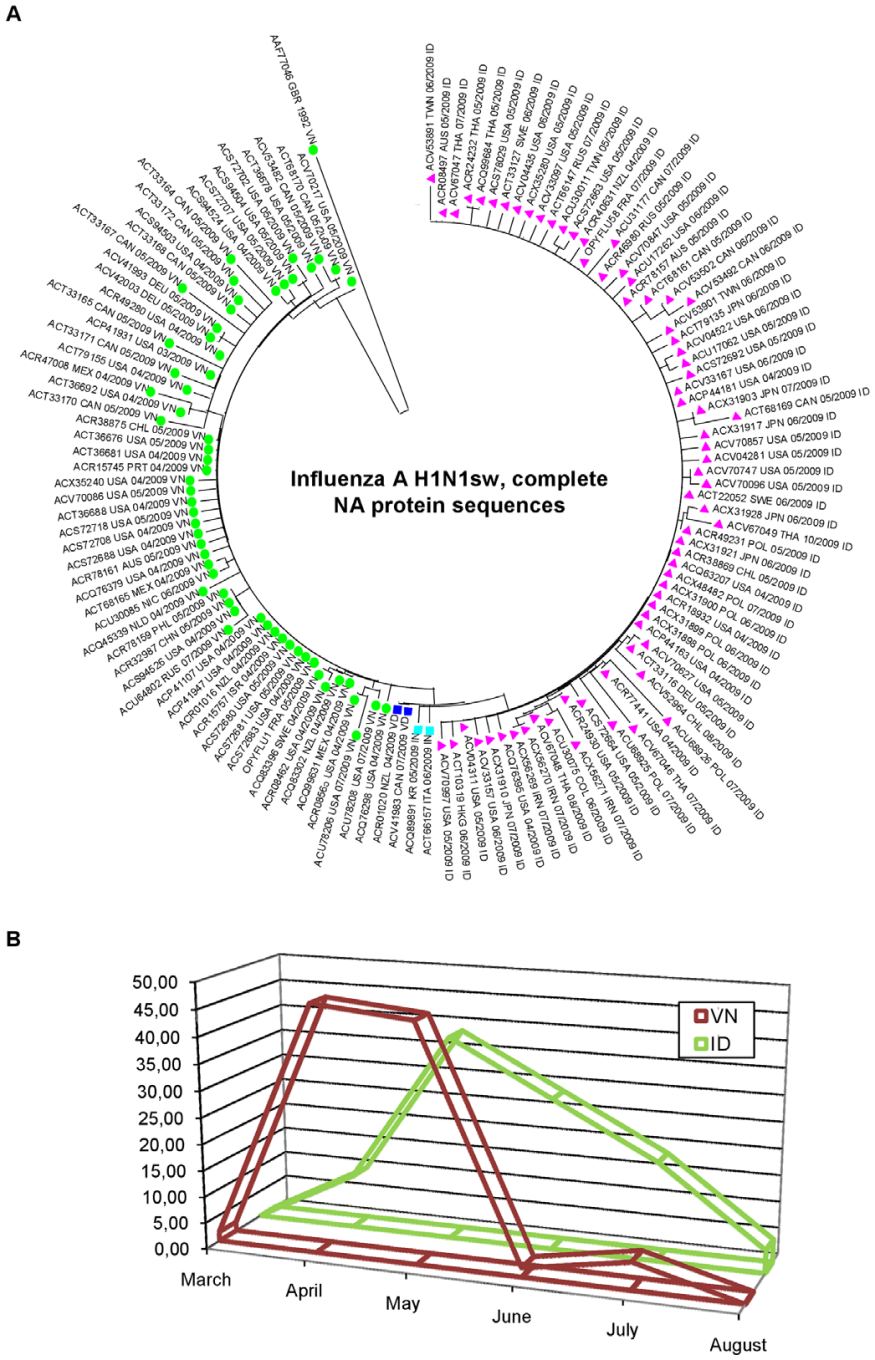
Phylogenetic and temporal distribution of H1N1sw isolates. Figure 10A shows the phylogenetic distribution of H1N1sw isolates based on complete amino acid neuraminidase sequences. The label of each strain includes the GenBank number, the country of origin, the time of collection and the amino acid pattern at residue positions 106 and 248 of the neuraminidase protein. Figure 10B shows the temporal distribution of strains harbouring the VN or ID amino acid pattern at residue positions 106 and 248 of the neuraminidase protein. Figure 10B: Green circle: strains with VN pattern. Pink triangle: strains with ID pattern. Dark blue square: strains with VD pattern. Light blue square: strains with IN pattern.

Comparative analysis of genetic amino acid distances amongst H1N1sw 2009 HA and NA sequences available in databases at mid-October 2009 revealed that the genetic diversity of protein sequences was minor, but slightly more notable in the NA gene (∼2% *vs* ∼1% in HA gene). OPYFLU-1 and OPYFLU-58 HA protein sequences were identical (with 3 synonymous substitutions) but 2 non-synonymous differences were detected in the NA gene (V106I, N248D) in addition to 3 non-synonymous mutations. There was no evidence of resistance to neuraminidase inhibitors in either strain. Interestingly, strains appeared to segregate according to the nature of residues 106 and 248. When using amino acid sequences, various methods used for tree building (including distance-based neighbor joining and maximum parsimony reconstructions) provided a similar topology, with VN strains appearing ancestral, separate clusters including VD and IN sequences and finally a large group of ID sequence that seemed to have emerged more recently from a common ancestor (see figure 10A). This chronology is globally validated by the analysis of dates at which the corresponding strains were collected (see figure 10B). However, bootstrap resampling values at forks delineating the main clusters are low (<50), a possible consequence of the limited genetic distances between the different strains studied. Analysis performed using nucleotide sequences similarly provided phylogenetic inconclusive results with similar grouping of strains according to their VN, VD, IN or ID pattern.

## Discussion

In a number of countries, the appearance of the H1N1sw pandemic resulted in reinforcement of the surveillance of influenza-like illnesses (ILIs). In countries located in the Northern hemisphere, this led to a follow up of ILIs during the spring and summer periods. Paradoxically, this revealed our limited knowledge concerning the epidemiology of viral respiratory diseases outside the winter epidemic season and contributed novel information regarding the new H1N1sw variant, but also seasonal influenza viruses, and other viral respiratory pathogens.

The study of the first 99 suspected cases identified in the French Southern Defence Zone showed that this population (mainly constituting adults 20–60 years old returning from abroad) was infected by a variety of respiratory viruses such as rhinoviruses, pneumoviruses, coronaviruses, enteroviruses, polyomaviruses, and parainfluenza viruses. Our analysis of this limited sample did not identify associations between age and specific pathogens. It provided results in agreement with those previously published by Follin and collaborators [23] and confirmed the difficult etiological identification of ILIs based on clinical presentation. One interesting aspect of this study was the identification of “seasonal” H3N2 influenza virus in June, July and August, which co-circulated at a low rate with the pandemic H1N1sw virus. This may appear to be an unusual feature in Southern France during summertime, but strongly suggests that such cases occur regularly and are just not detected by our standard surveillance system which focuses on investigations during the winter season. Therefore, the characteristics of the circulation of the influenza A virus may be much more complex than previously believed and our results suggest that the complete picture may include the circulation of “seasonal” virus during the April-August period in the Southern hemisphere, but also, at a low rate, in the Northern hemisphere: in our series, patients with H3N2 infections returned from Australia, but also from the UK or the USA.

Regarding H1N1sw infection, it was identified originally in travellers returning from abroad (75% in Panel A, including a majority of adults) or in patients in close contact with them (25%). This pattern was progressively modified and, notably, the number of patients who did not travel and could not identify any link with suspected cases, as well as the number of patients under the age of 10 tested for H1N1sw infection, increased progressively. The final picture (see figures 3B and 4) is very similar to the epidemiological distribution of H1N1 seasonal virus usually observed in age groups, with ∼50% of cases under the age of 20, a decreasing number of cases in age groups over the age of 20 and, notably, a very limited number of cases in patients over 60 years old. Superimposing the curves of empirical cumulative distribution of ages for patients with seasonal H1N1 in New York State during the 2006–2007 and 2007–2008 influenza seasons, and for H1N1sw in Panel C shows a striking similarity. Only one difference is observed in the 2–12 years old age group, in which the number of cases is slightly lower in our series. However, it must be noted that, in the current study, the investigation of clusters of cases (implicating a majority of children under the age of 15) was limited to sampling a few individuals *per* cluster. Therefore, the actual number of cases in children was underestimated and is most probably similar to that reported for seasonal H1N1 by [20] (in the latter study, 47% of the detected H1N1 cases were reported in patients younger than 20 years). The most remarkable difference observed by [20] between seasonal H1N1 (and thus H1N1sw) and H3N2 distribution, is the number of cases occurring in the elderly, (H1N1 does preferentially target a younger population). One reason that may underlie this difference is the weaker antigenic drift in H1N1, associated with co-circulation of multiple H1N1 lineages and weaker H1N1 bottleneck effects between seasons compared to those of H3N2 [20], [24]. Indeed, in the current series the number of cases detected in patients over 60 years old is remarkably low: less than 1% in both Panels B and C whilst this age group provided 10.5% of the patients tested in these two panels.

This distribution of cases in age groups is of special interest in the light of HI serological results (see figures 8 and 9): regardless of the antibody titre considered, it appears clear that the prevalence of antibodies to H1N1sw is low under the age of 30. Since it is extremely improbable that strains related to H1N1sw circulated in human populations during the last 20 or 30 years, the value observed for young patients is likely to be due to cross reactivity with seasonal influenza and thus indicative of the global overestimation of the prevalence provided by the HI assay (according to this hypothesis, around 10%). In individuals over 40, the prevalence is clearly disconnected from that observed for seasonal viruses and suggests previous exposure (presumably before 1970) to influenza virus(es) antigenically related to the current H1N1sw. However, much earlier circulation (*i.e.* before 1940 and possibly between the first and the Second World War) of H1N1sw-related strains cannot be ruled out considering the high prevalence values observed for patients over 80.

This suggests a “cause and effect” relationship, *i.e.* protection provided by specific antibodies. However, this interpretation should be considered tenuous since the significance of the titres of HI antibodies detected, in terms of protection against infection/ asymptomatic infection/severe forms, is unknown. Moreover, if the group of elderly individuals appears to be collectively prone to a low incidence of H1N1 and H1N1sw infections (at least of symptomatic infections), individuals without immunity to the virus do exist in this age group. Their precise number is unknown since the antibody level (HI titre) that may provide effective protection is undetermined, but the occurrence of a low incidence in this age group does not eliminate during the outbreak the risk of complicated forms and high mortality as classically observed in the case of seasonal influenza infection.

Concerning diagnosis of the acute infection, it is generally considered that the only reliable tool was the detection of viral genomes using molecular biological methods. Our comparative analysis of results obtained in parallel that incorporated molecular biology and a RIDT led to a more subtle assessment. We found that the positive predictive and specificity values of the RIDTs used were 100% and that the sensitivity in the age group 0–15 was 75%. Comparative analyses with studies investigating the performance of RIDTs suggest that the RIDT used in the current study performs better than others. This deserves further investigation. However, it also suggests that RIDT may be useful for rapid investigation of clusters of paediatric cases, and that they may also be particularly useful at the peak of the outbreak: we could calculate that (in the case of children under the age of 15 and under the hypothesis of a ratio of 2 between the price of the molecular test and that of the RIDT) the cost of a strategy associating a systematic RIDT and a molecular test for all negatives would become more attractive than systematically testing all samples by molecular biology for prevalence of influenza infection over 60%.

Another conclusion that could be drawn from the use of RIDTs is, in the case of H1N1sw, the more important viral excretion in children under the age of 15 compared with other age groups (see figure 6A). Actually, children were associated with the highest sensitivity of the test and, simultaneously, positive results of RIDTs could be associated with elevated viral loads (see figure 6B). This confirms previous results showing that the highest attack rates of seasonal influenza observed in communities of schoolchildren are accounted for by the shedding of higher titres of virus for a longer period than other patient groups [21], [22]. In the specific case of H1N1sw, similar results were observed suggesting that clinical attack rates in children under 15 years of age in La Gloria were twice those observed in adults [25].

The investigation of a cluster of cases in a summer camp showed the rapid spread of the virus in individuals living in the immediate vicinity of the index case. Interestingly, the attack rate observed in children (median age 11) was similar to that observed in young adults supervising them (median age 22). This strongly suggests that the shedding of higher titres of virus by children is the major parameter associated with high attack rates. However, it was also noticed that attack rates in children increased with age sub-groups, the risk of infection among teenagers being 3 times the risk of the youngest (under 8 years old). This difference may be, in the case of the current investigation, explained in part by the physical separation of children in different groups, but may also reflect specific behaviour or susceptibility to infection in different age groups.

Finally, the genetic characterisation of a strain isolated in the early period (May) of the outbreak from a patient returning from Mexico, and that of a strain isolated in July 2009 from a French autochthonous case revealed two mutations at positions 106 and 248 of the neuraminidase protein. Residue 106 is located at the N terminus of the neuraminidase domain and closely related to the trans-membrane domain. Residue 248 is located at the surface of the protein and part of an antibody recognition site [26]. It may therefore be associated with antigenic shift. The N248D mutation has previously been reported in H1N1 human strains isolated in the thirties, forties, seventies, eighties and nineties. Sequence analysis suggests that a majority of strains harboured the V^106^-N^248^ pattern at the origin of the outbreak. After May 2009, the decline of V^106^-N^248^ strains was concomitant with the emergence of I^106^-D^248^ strains. The mechanism of emergence of ID strains remains unclear: such strains may have emerged from a common ancestor and disseminated secondarily, but this hypothesis is epidemiologically puzzling and poorly supported by phylogenetic analyses in other genes such as HA. The alternative hypothesis (a common selection process may have lead to convergent evolution towards ID strains originating from various VN ancestors) cannot be ruled out and would suggest that evolutionary constraints led to the decline of V106-N248 strains after May 2009 and the emergence of I106-D248. To our knowledge, this phenomenon has not been associated to date with a change in the epidemiology or clinical presentation of the viral infection, but certainly deserves a careful follow up during the coming months.
